# A newly formed hexaploid wheat exhibits immediate higher tolerance to nitrogen-deficiency than its parental lines

**DOI:** 10.1186/s12870-018-1334-1

**Published:** 2018-06-07

**Authors:** Chunwu Yang, Zongze Yang, Long Zhao, Fasheng Sun, Bao Liu

**Affiliations:** Key laboratory of Molecular Epigenetics of Ministry of Education (MOE), Northeast Normal University, Changchun, 130024 China

**Keywords:** Wheat, Nitrogen uptake, Nitrate transporter, Allopolyploidy, Gene expression, Adaptation

## Abstract

**Background:**

It is known that hexaploid common wheat (*Triticum aestivum* L.) has stronger adaptability to many stressful environments than its tetraploid wheat progenitor. However, the physiological basis and evolutionary course to acquire these enhanced adaptabilities by common wheat remain understudied. Here, we aimed to investigate whether and by what means tolerance to low-nitrogen manifested by common wheat may emerge immediately following allohexaploidization.

**Results:**

We compared traits related to nitrogen (N) metabolism in a synthetic allohexaploid wheat (neo-6×, BBAADD) mimicking natural common wheat, together with its tetraploid (BBAA, 4*×*) and diploid (DD, 2*×*) parents. We found that, under low nitrogen condition, neo-6*×* maintained largely normal photosynthesis, higher shoot N accumulation, and better N assimilation than its 4*×* and 2*×* parents. We showed that multiple mechanisms underlie the enhanced tolerance to N-deficiency in neo-6*×*. At morphological level, neo-6*×* has higher root/shoot ratio of biomass than its parents, which might be an adaptive growth strategy as more roots feed less shoots with N, thereby enabling higher N accumulation in the shoots. At electrophysiological level, H^+^ efflux in neo-6*×* is higher than in its 4*×* parent. A stronger H^+^ efflux may enable a higher N uptake capacity of neo-6*×*. At gene expression level, neo-6*×* displayed markedly higher expression levels of critical genes involved in N uptake than both of its 4*×* and 2*×* parents.

**Conclusions:**

This study documents that allohexaploid wheat can attain immediate higher tolerance to N-deficiency compared with both of its 4*×* and 2*×* parents, and which was accomplished via multiple mechanisms.

**Electronic supplementary material:**

The online version of this article (10.1186/s12870-018-1334-1) contains supplementary material, which is available to authorized users.

## Background

Polyploidy, or whole genome duplication (WGD), is a pervasive driving force in the evolution of higher plants [1–4]. Polyploidy has also contributed significantly to the domestication of important crops such as wheat, canola, potato, sugarcane and cotton. Polyploidy in general and allopolyploidy (WGD of interspecific hybrids) in particular, can instantaneously induce genetic and epigenetic changes an altered gene expression at both total and homeolog-specific levels [5]. Some studies have documented that polyploidy may lead to immediate physiological and morphologic innovations such as increased photosynthetic capacity, changed flower colors, and enhanced tolerance to biotic and abiotic stresses [6–10], suggesting that the rapid genetic and epigenetic changes as well as altered physiological properties are likely consequential to adaptive evolution in polyploids.

Hexaploid common wheat (*Triticum aestivum* L*.*, genome BBAADD) is an very young allohexaploid species (ca. 8500 year-old) yet rapidly became one of the most important food crops worldwide, and is still so nowadays [11, 12]. Domestication of polyploid wheats symbols modern civilization in West Asia and Europe. The formation and success of hexaploid wheat also provides a suitable system to explore whether polyploidy-specific properties have evolved in the course of polyploid genome evolution or being conferred immediately following polyploidization because their progenitor species, tetraploid wheat (*T. turgidum*, genomes BBAA) and the D-genome goat-grass (*Aegilops tauschii*) are still extant.

Physiological systems of cultivated tetraploid wheat and wild *Ae. tauschii* have evolved independently under distinct selective pressures, and which conceivably have sculpted their physiological traits to different states. As such, they may have contrasting features of C and N metabolisms. It is therefore interesting to understand how N metabolism responds and behaves and whether or how the nitrogen use efficiency of hexaploid common wheat has changed immediately after merging and doubling the BA and D genomes by allohexaploidization. For this purpose, reconstructed synthetic allohexaploid wheat mimicking natural common wheat is the material of choice.

Plant roots absorb nitrate (NO_3_^−^) from soil using a larger transporter family which can be grouped into two classes: the NPF (nitrate transporter 1/peptide transporter family, NRT1) and high-affinity NRT2 gene family [13]. In *Arabidopsis thalian*, 53 NPF genes and seven NRT2 genes were identified [14, 15]. Hexaploid wheat may have much larger NPF and NRT 2 families because each member may include three or more homeologs. It is known that absorbed NO_3_^−^ by root is first reduced to nitrite by nitrate reductase (NR) and then to NH_4_^+^ by nitrite reductase (NiR). NH_4_^+^ is then incorporated into amino acids by glutamine synthetase (GS) and glutamate synthase (GOGAT) or the alternative glutamate dehydrogenase (GDH) pathway. In higher plants, photosynthesis, photorespiration and N assimilation form complex interactive networks: the GOGAT/GS cycle removes the toxic NH_4_^+^ derived from photorespiration to protect the photosynthetic enzymes, and photosynthesis supplies the GOGAT/GS cycle with reducing powers, in the forms of NADPH, ATP or Fdred [13–16]. Accordingly, the GOGAT/GS cycle, GDH, photorespiration, nitrate transporter and photosynthesis are core components for nitrogen metabolism regulation.

In order to investigate whether and by what means the nitrogen metabolism in hexaploid wheat is changed immediately after merging and doubling the BA and D genomes by allohexaploidization, we compared physiological traits relevant to nitrogen metabolism of a synthetic allohexaploid wheat (neo-6*×*; genome BBAADD) with its exact parental genotypes, *Ae. tauschii* (genome DD) (2*×*) and *T. turgidum* (genome BBAA) (4*×*) and analyzed gene expression under normal and low N conditions. A natural hexaploid wheat cultivar (nat-6*×*) was also included for the comparison. We show that allohexaploidization has instantaneously changed nitrogen metabolism traits in the synthetic hexaploid wheat.

## Results

### Growth and photosynthesis

**Fig. 1:**
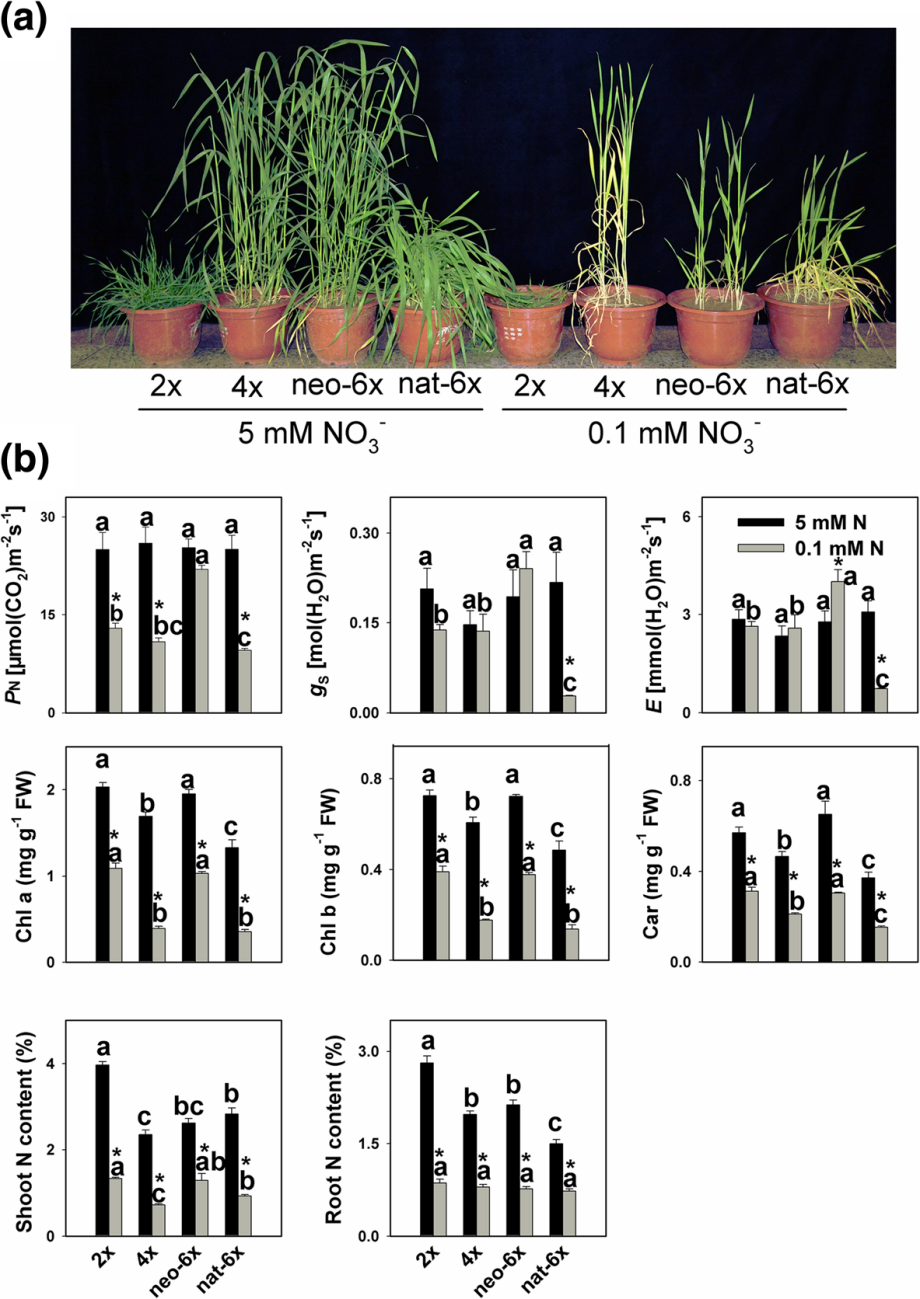
Effects of low N condition on growth, photosynthesis and nitrogen content in a newly formed hexaploid (neo-6*×*), its diploid (2*×*) and tetraploid (4*×*) parents, and natural allohexaploid (nat-6*×*). 17-day-old seedlings were subjected to low N condition (0.1 mM) for 31 days. The values are means of four biological replicates. **a** Growth status of four wheat lines under 0.1 mM and 5 mM N conditions. **b** Photosynthesis and nitrogen content: *g*_s_ − stomatal conductance; *P*_N_ − net photosynthetic rate; Chl – chlorophyll; Car–carotenoid; *E* − transpiration rate. Asterisks indicated significant difference (*t* test, *P* < 0.05) between control and low N-stressed plants for a given genotype. The means of any two of all four lines at the same N condition were compared using *t* test (*P* < 0.05), and means followed by different letters at the same N condition are significant

More than 98% of the total nitrogen in soil is in the form of organic matter, which however is mostly unavailable directly to plants. The organic N is transformed to available inorganic forms (ammonium, nitrite, and then nitrate) by soil microorganisms [16]. In typical aerobic agricultural soil, nitrate is the major inorganic N form. The usual concentrations of available inorganic N in agricultural soils range from 1 to 10 mM [16]. In this study, we set 5 mM of NO_3_^−^ as normal N condition, and 0.1 mM as a low N condition. All four wheat lines grew well under normal N condition, but under the low N condition, neo-6*×* had much stronger roots and better leaves than 4*×* (Fig. 1a and Additional file 1: Figure S1, Additional file 2: Figure S2). We measured three photosynthetic parameters, *P*_N_, g_S_, and *E* under both N conditions, and found that the four wheat lines were similar in all three parameters under normal N condition (Fig. 1); however, under low N condition, the three parameters were much higher in neo-6*×* than in its parental genotypes and, surprisingly, even higher than nat-6*×*. Chlorophyll a and chlorophyll b had similar responses to low N condition, and under both N conditions, neo-6× had higher chlorophyll and carotenoid contents than 4*×* and nat-6*×* but similar to 2*×*. In comparison, low N condition strongly reduced the accumulation of chlorophylls, but the decrements in neo-6*×* (chlorophyll a 47% and chlorophyll b 48%) were more moderate than those in 4*×* (chlorophyll a 77% and chlorophyll b 71%) and nat-6*×* (chlorophyll a 73% and chlorophyll b 72%) although similar to 2*×* (chlorophyll a 46% and chlorophyll b 46%). We also measured the main photosynthetic electron transport parameters: PSII efficiency (Φ_PSII_), efficiency of excitation capture by open PSII centers (F_v_’/F_m_′), and maximum quantum yield of photosystem II (F_v_/F_m)_ (Additional file 3: Figure S3). Results showed that low N slightly reduced the Φ_PSII_ in all four wheat lines, but the decrements in nat-6*×* and neo-6*×* were to less extents than 2*×* and 4*×*. Low N only had a moderate effect on F_v_’/F_m_′ and F_v_/F_m_. These data revealed that the central photosystem was not damaged by the low N condition. Under normal N, N content is significantly higher in 2*×* than in 4*×*, while N content in neo-6*×* was only slightly higher than in 4*×* (Fig. 1b, *P* > 0.05). In shoots, the low N significantly decreased N accumulation in all four wheat lines, but again the decrement in neo-6*×* (51%) was smaller than those of 2*×* (66%), 4*×* (70%) and even nat-6*×* (67%) (Fig. 1b). Under low N, shoot N content in neo-6× was 1.79 folds of that of 4*×*, but there was no difference in root N content. Taken together, neo-6*×* had smaller reduction in *P*_N_ and shoot N content than the other three wheat lines, indicating that neo-6*×* has stronger ability for N accumulation than 2*×* and 4*×* under the low N condition. To test generality of this finding, we also compared low N responses in additional four tetraploid wheat lines and two synthetic hexaploid wheat lines. The results showed that all the tetraploids had much higher chlorophyll and carotenoid reductions than the neo-6*×* lines (Additional file 4: Figure S4).

### Nitrogen assimilation

**Fig. 2:**
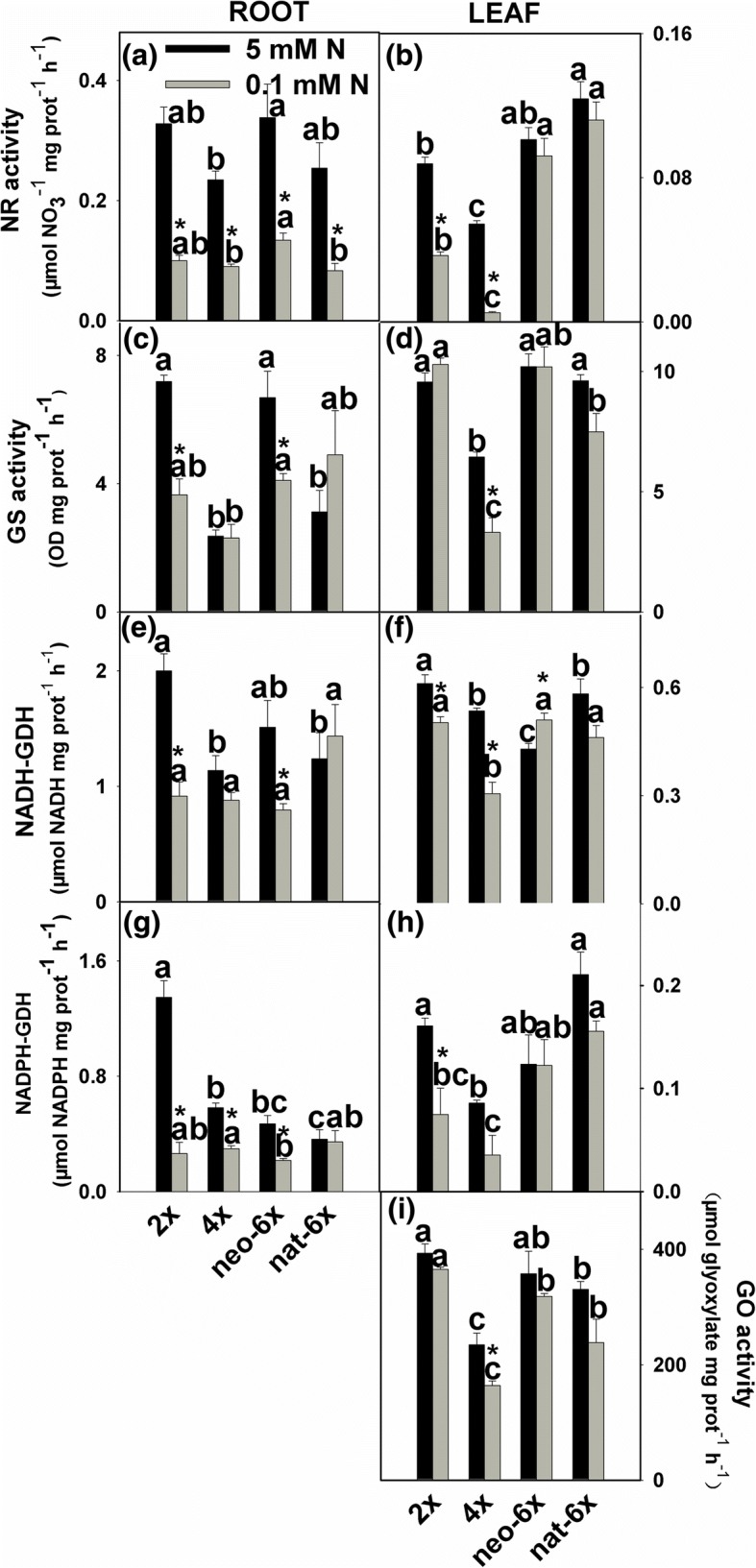
Effects of low N condition on activities of enzymes involved in nitrogen assimilation in a newly formed hexaploid (neo-6*×*), its diploid (2*×*) and tetraploid (4*×*) parents, and natural allohexaploid (nat-6*×*). The enzymes in the fresh mature leaves at the same leaf position for each wheat line were assayed. Ten mature leaves from five individual plants for each wheat line was pooled as a biological replicate. The values are means of four biological replicates. Asterisks indicated significant difference (*t* test, *P* < 0.05) between control and low N-stressed plants for a given genotype. The means of any two of all four lines at the same N condition were compared using *t* test (*P* < 0.05), and means followed by different letters at the same N condition are significant. The seedlings were subjected to low N condition (0.1 mM) for 7 days. NR, nitrate reductase; GS, glutamine synthetase; GDH, glutamate dehydrogenase; and GO, Glycolate oxidase

**Fig. 3:**
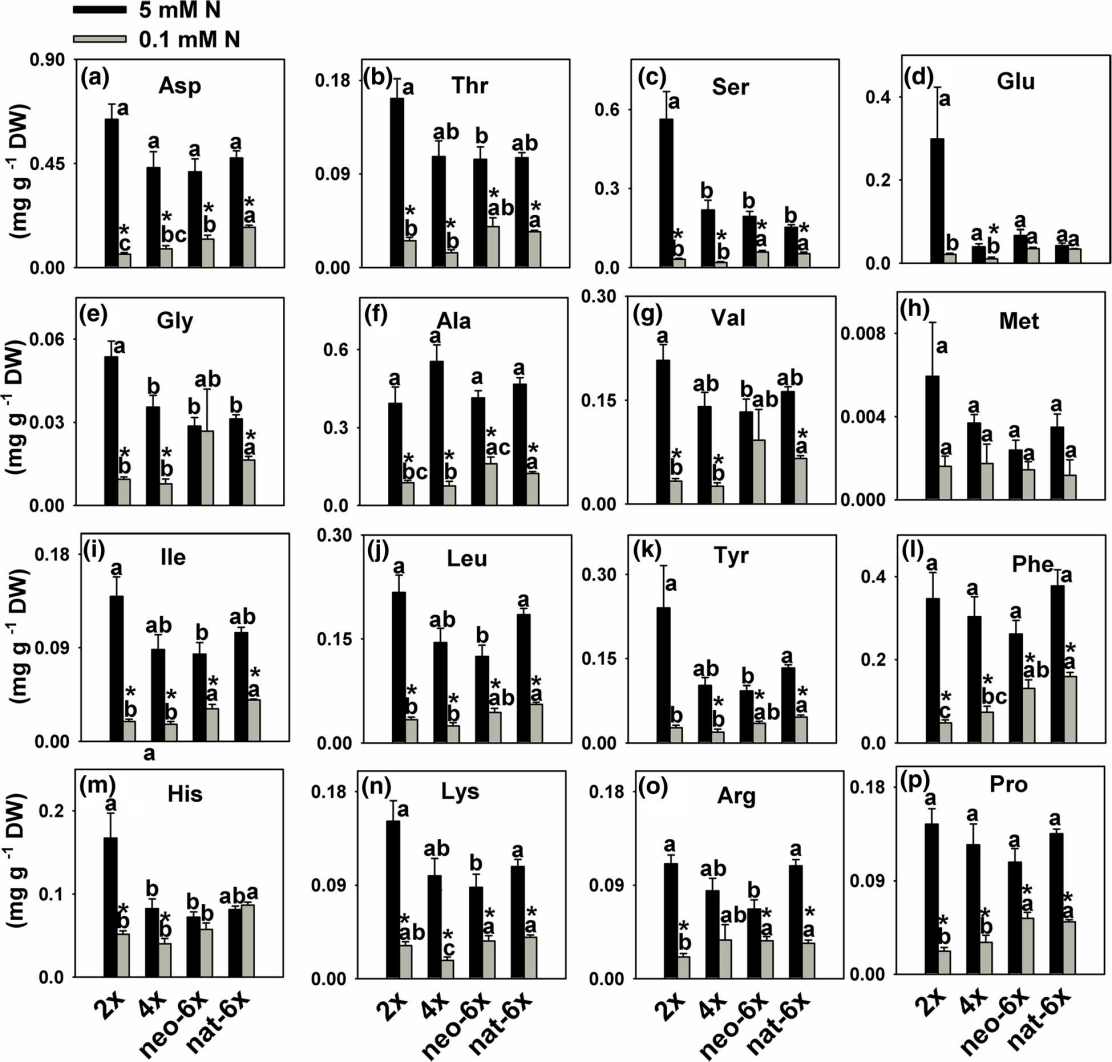
Effects of low N condition on the contents of amino acids in shoots of a newly formed hexaploid (neo-6*×*), its diploid (2*×*) and tetraploid (4*×*) parents, and natural allohexaploid (nat-6*×*). The values are means of four biological replicates. Asterisks indicated significant difference (*t* test, *P* < 0.05) between control and low N-stressed plants for a given genotype. The means of any two of all four lines at the same N condition were compared using *t* test (*P* < 0.05), and means followed by different letters at the same N condition are significant. The seedlings were subjected to low N condition (0.1 mM) for 31 days

We measured activities of most key enzymes involved in nitrogen assimilation, and activity of glycolate oxidase (GO), a rate-limiting enzyme for photorespiration. We first compared the differences among the four wheat lines under normal N condition. Results showed that, under normal N (5 mM) condition, 2*×*, neo-6*×* and nat-6*×* all exhibited higher values than 4*×* in activities of NR GS, NADPH-GDH and GO in leaves; 2*×* and neo-6*×* have higher NADH-GDH, NR and GS activities than 4*×* and nat-6*×* in roots (Fig. 2). In leaves, low N condition greatly reduced the NR activity in 2*×* and 4*×* but not in neo-6*×* and nat-6*×* at the 7-day time-point (Fig. 2). Low N only reduced the GS activity in 4*×* leaves, but not in the other three wheat lines at both time points (Fig. 2 and Additional file 5: Figure S5). In roots, when plants were grown under low N condition for 7 days, NADH-GDH activities decreased in 2*×* and neo-6× but not in 4*×* and nat-6*×*, and NADPH-GDH activities decreased in 2*×*, 4*×* and neo-6*×* but not in nat-6*×*. In leaves, NADH-GDH activities were reduced in 2*×*, 4*×* and neo-6*×* but not in nat-6, while NADPH-GDH activity was only decreased in 2*×* (Fig. 2). Under low nitrogen condition, glycolate oxidase (GO) activities of all four wheat lines showed decreasing tendency, but only 4*×* reached statistical significance and the decrement was greater than neo-6*×* (Fig. 2). We did not detect the activities of GO in roots of all four wheat lines. Under normal N condition, in leaves, contents of all the measured amino acids in neo-6*×* were similar to 4*×*; however, under low N condition, contents of most amino acids were lower in 4*×* than neo-6*×* (Fig. 3). Comparing the decrements of most amino acids, by low N condition, neo-6× showed lower values than 2*×* and 4*×* (Additional file 6: Figure S6). In roots, 10 of 16 amino acids showed that both neo-6*×* and 2*×* had greater decrease than 4*×* (Additional file 7: Figure S7, Additional file 8: Figure S8). In summary, neo-6*×* could maintain a higher nitrogen assimilation level than 2*×* and 4*×* under low N condition only in leaves but not in roots. In roots, effect of low N on neo-6*×* nitrogen assimilation was similar to 4*×* even neo-6*×* accumulated less amino acids. Taken together, it is clear that, under normal N condition, diploid parent of the newly formed hexaploid wheat exhibited a much higher N accumulation, NR and GS activities, and chlorophyll contents than the tetraploid parent. This suggests that they might have different N metabolism features. In low N stressed leaves, neo-6*×* could maintain a relatively normal N assimilation status and produce more assimilated N products than 2*×* and 4*×*; however, in roots, neo-6× had similar N assimilation as 2*×* and 4*×*.

### Nitrate uptake and expression of NPF and NRT genes

**Fig. 4:**
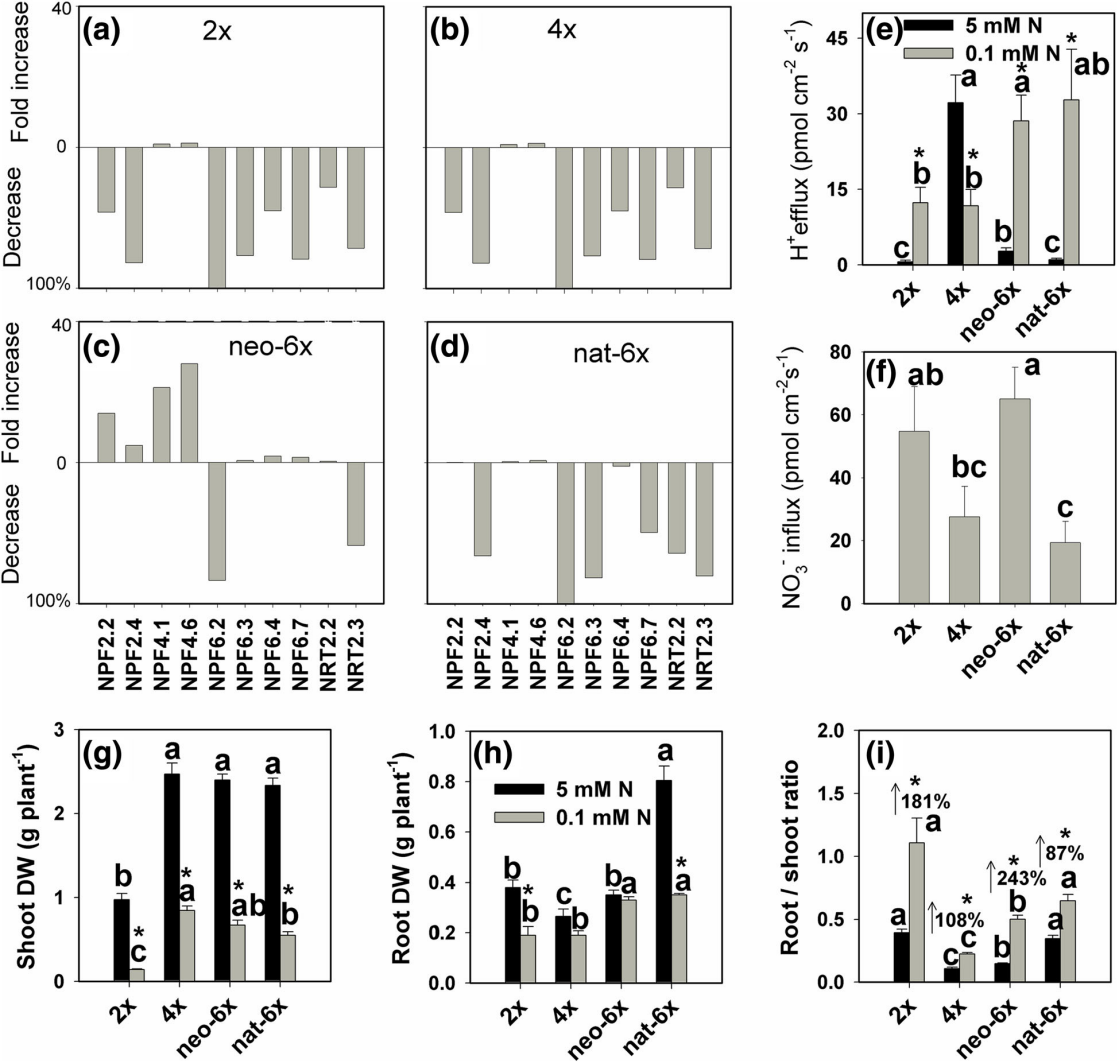
Effects of low N condition on the expression of root nitrate transporter genes (**a-d**), H^+^ efflux (**e**), NO_3_^−^ influx (**f**), dry weight (DW) (**g-h**) and root/shoot ratio (**i**) in a newly formed hexaploid (neo-6*×*), its diploid (2*×*) and tetraploid (4*×*) parents, and natural allohexaploid (nat-6*×*). (**a-d**) The fold increase of the gene expression was calculated according to (treatment-control) /control, and the percentage of gene expression decrease was calculated according to (control-treatment)*100% /control. (**e-i**) Asterisks indicated significant difference (*t* test, *P* < 0.05) between control and low N-stressed plants for a given genotype. The means of any two of all four lines at the same N condition were compared using *t* test (*P* < 0.05), and means followed by different letters at the same N condition are significant. The values are means of 3–7 biological replicates. When the seedlings were subjected to low N condition (0.1 mM) for 7 days, gene expression, NO_3_^−^ influx and H^+^ efflux were measured. The dry weights of the roots and shoots were measured and root DW /shoot DW ratio (root /shoot ratio) was calculated at 31 days of the stress. Under 5 mM N condition, we did not detect any NO_3_^−^ flux signal due to high background signal of NO_3_^−^, thus, NO_3_^−^ influx data of only 0.1 mM N treatment was displayed in the Figure **f**

We observed that low N stress dramatically enhanced H^+^ effluxes of roots in 2*×*, nat-6*×* and neo-6*×*, but decreased H^+^ efflux of roots of 4*×* (Fig. 4). Under low N condition, H^+^ effluxes were higher in neo-6*×* and nat-6× than in 2*×* and 4*×*. We also investigated the NO_3_^−^ fluxes in roots under both N conditions. Unfortunately, under 5 mM N condition, we did not detect any NO_3_^−^ flux signal due to high background of NO_3_^−^. Thus, we only measured NO_3_^−^ flux under the low N condition (Fig. 4f). Results showed that NO_3_^−^ influx in neo-6*×* was much higher than those in 4*×* and nat-6*×* under low N condition (Fig. 4f). Under low N condition, shoot dry weight (DW) of neo-6*×* appeared slightly lower than that of 4*×*, although the difference was not significant statistically, whereas root DW was much higher in neo-6*×* than those in 4*×* and 2*×* (Fig. 4g-h). Root/shoot ratios of DW were elevated in low N condition in all the four lines, but the enhanced degree was higher in neo-6*×* than those in the other three wheat lines (Fig. 4i). Root /shoot ratio of DW in neo-6× was much higher than that in 4*×* under low N condition (Fig. 4i)**.** We next measured the expression of eight NPF genes and two NRT2 genes in roots. Results showed that low N condition down-regulated the expression of *NPF6.3* and *NPF 6.7* in 2*×*, 4*×* and nat-6*×* but slightly up-regulated the expression of both genes in neo-6*×* (Fig. 4a-d and Additional file 9: Figure S9). Remarkably, the low N condition enhanced *NPF4.6* expression in neo-6*×* by 28-fold, while the induction of this gene expression in the other three wheat lines was only within the range of 0.59–1.9-fold. Similarly, low N condition elevated the expression of *NPF4.1* in neo-6*×* by 21.3-fold, but only in the range of 0.24–2.35-fold in the other three wheat lines (Fig. 4 and Additional file 9: Figure S9).

## Discussion

**Fig. 5:**
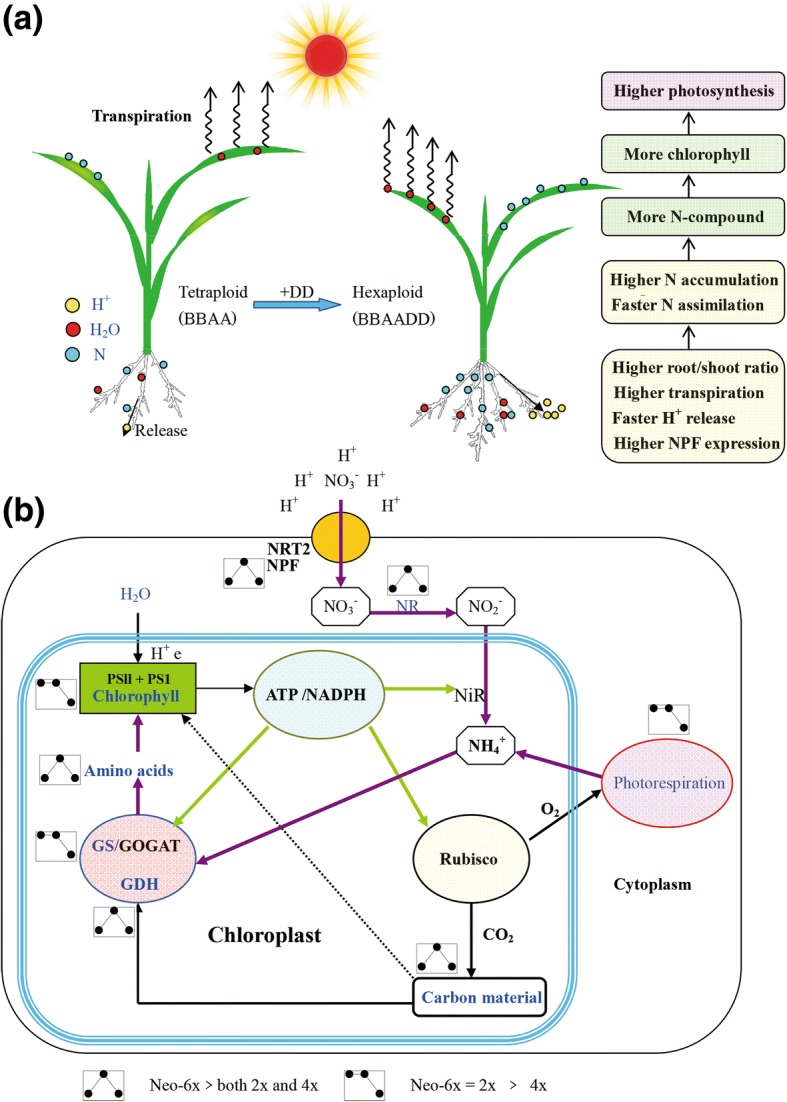
Nitrogen-deficiency tolerance mechanism of a newly formed hexaploidy (neo-6*×*). **a** Comparative characteristics of H^+^, H_2_O and NO_3_^−^ uptakes of a newly formed hexaploid (genome BBAADD) and its tetraploid (genome BBAA) parent under low N condition. **b** Differences between a newly formed hexaploid (neo-6*×*) and its diploid (2*×*) and tetraploid (4*×*) parents in photosynthesis and nitrogen metabolism under low N condition

Nitrogen is one of the staple nutrients for crops, and involved not only in seed production but also in the responses to stressful conditions. Globally, in order to feed the ever-increasing human population, almost 10^11^ kg of N per annum is applied to the agroecosystem [16, 17]. However, the crops are able to utilize only 30–40% of the applied N [16, 17]. The loss of N not only elevates cost of agricultural production, but also aggravates soil and water pollution [18]. Thus, crop cultivars with high nitrogen uptake efficiency that can grow reasonably well at low N condition are of great importance for decreasing N input to the agroecosystem [18, 19]. Our results of this study have shown that traits related to N metabolism in polyploid wheat is associated with their genome compositions as far as a synthetic hexaploid wheat (analogous to common wheat in genome composition) is concerned. Under normal N condition, shoot N and amino acid contents, NR and GS activities as well as chlorophyll contents are all higher in neo-6*×* than in 4*×*. This suggests that the different soil N conditions experienced by the 2*×* and 4*×* probably have shaped their N metabolic efficiencies. After merging and doubling the AB and D genomes by allohexaploidization (leading to speciation of *Triticum aestivum* L.), nitrogen metabolism has been synergistically enhanced probably by the positive epistatic interactions between the BBAA and DD subgenomes. Interestingly, we observed that, under low N condition, neo-6*×* can maintain relatively normal photosynthesis, higher shoot N accumulation, and better N assimilation status than its 2*×* and 4*×* parents (Fig. 5). This suggests that following allohexaploidization, hexaploid wheat can immediately achieve a stronger N-deficiency tolerance than its 2*×* and 4*×* parents. However, we should caution that we have compared only one nat-6× and limited lines of neo-6*×*. Nevertheless, we consider this observation is interesting in that if generally confirmed, it may suggest that due to persistent over-dose application of N in modern agricultural practice, the otherwise much stronger N utilization efficiency immediately acquired by newly formed hexaploid wheat has been eroded. We consider that this matter merits further studies, as it may have significant implications to employing the synthetic wheat strategy in wheat genetic improvement [20].

Previous studies have also indicated that polyploidy can instantaneously enhance stress tolerance of plants, and the enhanced tolerance is considered to be related to root feature innovation. For example, in *Arabidopsis* tetraploid, it was documented that higher K accumulation and salt tolerance of the tetraploid are controlled by root ploidy, independently of the ploidy of the shoot [8]. In a newly formed hexaploidy wheat, the stronger capacity of root Na^+^ control contributes its salt tolerance [10]. However, little is known about the timing of emergence for the adaptive traits of hexaploid wheat. Our study suggests that major physiological characteristics underlying N-deficiency tolerance of a newly formed hexaploid wheat can form immediately following allopolyploidization. First, at morphological level, neo-6*×* has higher root/shoot ratio in biomass under low N condition. It may be an adaptive growth strategy for neo-6*×* as more roots feed less shoots with N, thereby enabling much higher N accumulation in the shoots. Higher transpiration rate of neo-6*×* may also facilitate the long-distance transport of N-compounds from its roots to shoots because transpiration is main driving force for ion and water uptake. Second, at electrophysiological level, we measured the H^+^ flux because H^+^ gradient or H^+^ motive force is the energy driving NO_3_^−^ uptake by roots. Usually, low N stress dramatically enhance H^+^ efflux of roots to elevate the uptake of NO_3_^−^ [21]. Our results also showed that, under low N stress condition, 2*×* parent, neo-6× and nat-6× all have an increasing tendency for H^+^ efflux; however, 4*×* parent showed a decreasing tendency. Under low N condition, H^+^ efflux in neo-6*×* was higher than their parents. These data suggested that the stronger H^+^ efflux of neo-6*×* may have contributed its stronger N uptake capacity. Indeed, NO_3_^−^ influx of neo-6*×* was much higher than that of 4*×* under low N condition. Finally, at gene expression level, we assayed the expression of NPF or NRT2 gene families in the roots because they are dominant NO_3_^−^ transporters [13], over a wide range of external NO_3_^−^ concentrations [22, 23]. The results showed that neo-6*×* has significantly higher expression levels in several NPF genes than both parents. Especially, low N condition up-regulated *NPF4.1* and *NPF4.6* expression in neo-6*×* by 21.3- and 28-fold, respectively, while only slightly enhanced their expression levels in 2*×* and 4*×* parents. In addition, low N stress down-regulated *NPF2.2* and *NPF2.4* in 2*×* and 4*×* parents, but up-regulated their expression in neo-6*×*, exhibiting an immediate expression reprogramming from low N-inhibitive expression of 2*×* or 4*×* parent to -induced expression of neo-6*×*. Under a limiting N condition, the higher expression levels of NO_3_^−^ transporter genes may enable N-uptake faster in the newly formed hexaploid wheat.

## Conclusions

Nitrogen metabolism is critical for growth, development and adaptive responses of plants (Fig. 5). Nitrogen metabolism alterations of plants may affect multiple metabolism processes such as photosynthesis, photorespiration and secondary metabolisms. Nitrate uptake, NR and GOGAT/GS cycle are three core components for N metabolism, which drives N assimilation and down-stream metabolism processes (Fig. 5). We propose that the newly formed hexaploid displayed much higher N-uptake and -assimilation efficiencies and greater root biomass compared with its tetraploid parent under a N-limiting condition, which may be achieved by the incorporated DD genome, fixed heterosis or other mechanisms. In this work, we document major biochemical and gene expression regulation mechanisms underlying high N-uptake and -assimilation efficiencies of the synthetic allohexaploid wheat (Fig. 5). From an evolutionary perspective, our work may improve the understanding of the mechanisms regulating stronger adaptability of hexaploid wheat. We found that the stronger N-deficiency tolerance of the synthetic hexaploid wheat was attributable to its novel features of root. Together with previous studies, it suggests that innovation of root feature may be a critical component for adaptive evolution of polyploids, which may have implications for the breeding of polyploid crops with enhanced abiotic stress tolerance.

## Methods

### Plant material

In this work, we used a newly formed allohexaploid wheat line (Allo-960, genome BBAADD), its two parents, and a natural hexaploid wheat (cv. Chinese Spring) as main experimental organisms. Allo-960 was produced by crossing a *Triticum turgidum* line (Black bird, genome BBAA) with a *Aegilops tauschii* line (30A, genome DD) [10]. The seeds of Allo-960 and its parents were provided from George Fedak (Agriculture and Agri-Food in Canada). In this study, sixth self-pollinated generations of Allo-960 was used and labeled as neo-6*×*, its tetraploid parent as 4*×*, its diploid parent as 2*×*, and the natural hexaploid wheat (cv. Chinese Spring) as nat-6*×*. In this work, to explore the physiological mechanisms of N-deficiency tolerance alteration of the newly formed hexaploid, we utilized Allo-960 and its exact 2*×* (30A) and 4*×* (Black bird) parents to measure gene expression and physiological indices. To test generality of the results, we also used another *Ae. Tauschii* line, two newly formed hexaploid lines, and 4 tetraploid lines, but we only measured their chlorophyll contents to indicate the N-deficiency tolerance alteration. All wheat lines used were listed in Additional file 10: Table S1.

### Stress treatment

According to the typical concentration of available inorganic N in agricultural soils (1 to 10 mM) [24–26], we set 5 mM of NO_3_^−^ as control N condition, and 0.1 mM as a low N stress. Seeds of all wheat lines were sown in pots containing thoroughly washed sand. Each pot contained 5 seedlings as a biological replication. All seedlings were placed in a greenhouse with a thermoperiod of 20–23/13–17 °C and a 16/8 h day/night photoperiod. The pots were watered with an altered half-strength Hoagland nutrient solution (control, 5 mM NO_3_^−^, pH 6.5) for 17 days before stress treatment. After which (i.e., at about the four-leaf stage), low N stress was applied for 7 or 31 days by supplementing nitrogen-deficient half-strength Hoagland’s solution (0.1 mM NO_3_^−^, pH 6.5). CaCl_2_ were used to compensate Ca^2+^ in low N treatment solution.

### Measurement of physiological indices

After 31 days of low N stress, net photosynthetic rate (*P*_N_), stomatal conductance (*g*_s_), and transpiration rate (*E*), PSII efficiency (ΦPSII), efficiency of excitation capture by open PSII centres (Fv’/Fm′) and maximum quantum yield of photosystem II (Fv/Fm) were determined using a portable open flow gas exchange system LI-6400 (LI-COR, USA) according to the protocol of the instrument. The photosynthetically active radiation (PAR) was 1200 μmol m^− 2^ s^− 1^. In order to minimize the plant-to-plant variations, five seedlings were pooled as a biological replicate, and there were at least four replicates for all biochemical measurements. Chlorophyll contents were determined according to the method reported in Ni et al. 2008 [27]. Free amino acids of dry sample were separated and measured by an automated amino acid analyzer [28]. Total nitrogen content was measured by an elemental analyser (Vario EL Cube, Elementar, Germany).

### Enzyme activity assays

The enzymes in the fresh mature leaves at the same leaf position for each wheat line were assayed using conventional methods. Ten mature leaves from five individual plants was pooled as a biological replicate, and there were four biological replicates. The activities of nitrate reductase (NR: EC 1.6.6.1), glutamine synthetase (GS: EC 6.3.1.2), and glutamate dehydrogenase (NADH-GDH: EC 1.4.1.2 and NADPH-GDH: EC 1.4.1.4) were measured according to altered methods of Debouba et al. 2006 and Surabhi et al. 2008 [29, 30]. Glycolate oxidase (GO: EC 1.1.3.15) were assayed with the method of Wu et al. 2013 [31]. Fresh plant tissues were used to determine activities of each enzyme. NR was extracted with 1 mL of buffer solution (100 mM KH_2_PO_4_-NaOH buffer of pH 7.4, 7.5 mM cysteine, and 1 mM EDTA, 1.5% casein) at 4 °C. 0.1 mL of the supernatant was incubated in a reaction mixture containing 0.3 mL 100 mM KH_2_PO_4_-NaOH buffer (pH 7.4), 0.1 mL 3 mM NADH, and 0.1 ml 100 mM KNO_3_ at 30 °C for 30 mins. The reaction was stopped by adding a stopping solution including 0.25 mL sulfanilamide, 0.25 mL 0.02% N-(1-naphthy) ethylenediamine dihydrochloride, and 0.1 mL glacial acetic acid. Finally, the absorbance was measured at 540 nm. The NR activity was expressed in μmol NO_3_^−^ mg protein^− 1^ h^− 1^. GS was extracted with 1 mL of buffer solution containing 25 mM Tris-HCl (pH 7.6), 1 mM MgCl2, 1 mM EDTA, 14 mM beta-mercaptoethanol, and 1% PVP at 4 °C. 0.1 mL of supernatant was added to a reaction mixture containing 0.3 mL 250 mM imidazole-HCl buffer (pH 7.0), 0.2 mL 300 mM L-glutamic acid sodium, 0.2 mL 30 mM ATP-Na_2_, and 0.1 mL 500 mM MgSO_4_ at 25 °C for 10 mins. The reaction was stopped by adding 0.1 mL 1 M hydroxylamine hydrochloride, 0.4 mL of a solution (3.3% FeCl_3_, 8% TCA and 17%HCl), and rotating for 10mins. Finally, the absorbance of reactive mixture was measured at 540 nm. The GS activity was expressed as OD at 540 nm mg protein^− 1^ h^− 1^. Glutamate dehydrogenase (NADH- and NADPH-specific GDH) was extracted with 1 mL of extraction buffer (100 mM pH 8.2 Tris-HCl buffer, 14 mM beta-mercaptoethanol, and 1% PVP) at 4 °C. 0.5 mL of supernatant was added to a reaction mixture containing 0.6 mL of 0.2 M Tris-HCl buffer (pH 8.0), 0.15 mL 0.1 M α-ketoglutarate sodium, 0.15 mL 1 M NH_4_Cl, and 0.1 mL 3 mM NADH (NADPH). The reaction was followed by measurement of the decrease in absorbance at 340 nm. The activities were expressed as μmol NADH mg protein^− 1^ h^− 1^ or μmol NADPH mg protein^− 1^ h^− 1^. GO was extracted with 1 mL of extraction buffer containing 100 mM KH_2_PO_4_-NaOH buffer (pH 8.0) at 4 °C. 0.05 mL of supernatant was incubated in a reaction mixture containing 0.5 mL 100 mM KH_2_PO_4_-NaOH buffer (pH 8.0), 0.1 mL 1 mM FMN, and 0.1 mL 50 mM glycollic acid for 5mins. The reaction was stopped by adding 0.1 mL 2 M HCl, 0.1 mL 1.82 M NaOH, 0.33% phenylhydrazine hydrochloride, and 1 mL concentrated HCl. The absorbance was measured at 550 nm. The GO activity was expressed in μmol glyoxylate mg protein^− 1^ h^− 1^.

### Measurement of H^+^ and NO_3_^−^ fluxes

The seeds were sown and germinated in 8.5-cm Petri dishes for 3 days. The dishes were placed in a growth room maintained at 25 °C day and 18 °C night temperatures under 16 h light at 300 μmol m^− 2^·s^− 1^.Then the young seedlings were transferred to a Petri dish containing the half-strength Hoagland nutrient solution with 0.1 or 5 mM NO_3_^−^ (pH 6.5) for 7 days. After 7 days of low N stress, net NO_3_^−^ and H^+^ fluxes at the surface of root maturation zone were measured using non-invasively scanning ion-selective electrode technique (SIET, SIET system BIO-003A, Younger USA Science and Technology, Falmouth, MA, USA) by Xuyue Science and Technology (Beijing, China). Six plants were randomly selected from each genotype and the roots were transferred to a Petri dish containing 10 ml the measuring solutions with 0.1 or 5 mM NO_3_^−^, and equilibrated for 2 h. Then the roots were transferred to a new Petri dish containing fresh measuring solutions with 0.1 or 5 mM NO_3_^−^, and net H^+^ and NO_3_^−^ fluxes were monitored for 8 min. The measuring solution of H^+^ flux composed of 0.1 mM KCl and 0.1 mM CaCl_2_ in pH 6.0, and the measuring solution of NO_3_^−^ flux composed of 0.1 mM CaCl_2_ and 0.3 mM MES in pH 6.0.

### Real time qRT-PCR

When the plants were grown at low N condition for 7 days, the leaves and roots were harvested to extract total RNA using TRIzol reagent (Invitrogen). Five individual plants of neo-6× were considered as five biological replicates, and for the other three lines the three plants were pooled as a biological replicate with 3–4 biological replicates. The RNA was treated with DNaseI (Invitrogen), reverse-transcribed using SuperScriptTM RNase H-Reverse Transcriptase (Invitrogen), and then subjected to qRT-PCR analysis using gene-specific primers (Additional file 11: Table S2). *Actin*, *RLI* and *GAPDH* were used as normalization control genes in the assay [32–34]. The expression of NPF genes and NRT2 genes was assayed with the primer sequences from previous studies [34, 35], and was calculated using △△Ct method [36].

### Statistical analysis

Statistical analysis was performed using the statistical program SPSS 13.0 (SPSS, Chicago, USA). All data were from 3 to 7 biological replicates. Statistical significance was determined by *t-*test.

## Additional files

Additional file 1: **Figure S1.** Effects of low N condition on growth status of a newly formed hexaploid (neo-6*×*), its diploid (2*×*) and tetraploid (4*×*) parents, and natural allohexaploid (nat-6*×*). The seedlings were subjected to low N condition (0.1 mM) for 28 days. (JPG 749 kb)

Additional file 2: **Figure S2.** Effects of low N condition on second leaf of a newly formed hexaploid (neo-6*×*), its diploid (2*×*) and tetraploid (4*×*) parents, and natural allohexaploid (nat-6*×*). The seedlings were subjected to low N condition (0.1 mM) for 28 days. Second leaf at below showed a clear difference among the four wheat lines under low N condition. (TIF 7026 kb)

Additional file 3: **Figure S3.** Effects of low N condition on photosynthetic electron transport in a newly formed hexaploid (neo-6*×*), its diploid (2*×*) and tetraploid (4*×*) parents, and natural allohexaploid (nat-6*×*). The seedlings were subjected to low N condition (0.1 mM) for 31 days. F_v_’/F_m_′, efficiency of excitation capture by open PSII centers; F_v_/F_m,_ maximum quantum yield of photosystem II_._ Asterisks indicated significant difference (*t* test, *P* < 0.05) between control and low N-stressed plants for a given genotype. (TIF 859 kb)

Additional file 4: **Figure S4.** Effects of low N condition on the chlorophyll and carotenoid contents of synthetic hexaploid wheats (BBAADD genome), diploid wheat (DD genome) and tetraploid wheats (BBAA genome). Diploid wheat:TQ18; newly formed (synthetic) hexaploid wheats: AT5, Allo-960 and ELI 13; tetraploid wheats: 37A, ALTAR81, black bird, BOT and TTR04. The seedlings were subjected to low N condition (0.1 mM) for 31 days. The values are means of three biological replicates. Asterisks indicated significant difference (*t* test, *P* < 0.05) between control and low N-stressed plants for a given genotype. (TIF 1779 kb)

Additional file 5: **Figure S5.** Effects of low N condition on the activities of enzymes involved in nitrogen assimilation of a newly formed hexaploid (neo-6*×*), its diploid (2*×*) and tetraploid (4*×*) parents, and natural allohexaploid (nat-6*×*). The values are means of four biological replicates. Asterisks indicated significant difference (*t* test, *P* < 0.05) between control and low N-stressed plants for a given genotype. The means of any two of all four lines at the same N condition were compared using *t* test (*P* < 0.05), and means followed by different letters at the same N condition are significant. The seedlings were subjected to low N condition (0.1 mM) for 31 days. NR, nitrate reductase; GS, glutamine synthetase; GDH, glutamate dehydrogenase; and GO, Glycolate oxidase. (TIF 1582 kb)

Additional file 6: **Figure S6.** Percent change of amino acids of the shoots under low N condition compared to control condition. The percentage was calculated according to (control-treatment)*100%/control. The seedlings of a newly formed hexaploid (neo-6*×*), its diploid (2*×*) and tetraploid (4*×*) parents, and natural allohexaploid (nat-6*×*) were subjected to low N condition (0.1 mM) for 31 days. (TIF 4881 kb)

Additional file 7: **Figure S7.** Effects of low N condition on the contents of amino acids in roots of a newly formed hexaploid wheat (neo-6*×*), its diploid (2*×*) and tetraploid (4*×*) parents, and natural allohexaploid (nat-6*×*). The values are means of four biological replicates. Asterisks indicated significant difference (*t* test, *P* < 0.05) between control and low N-stressed plants for a given genotype. The means of any two of all four lines at the same N condition were compared using *t* test (*P* < 0.05), and means followed by different letters at the same N condition are significant. The seedlings were subjected to low N condition (0.1 mM) for 31 days. (TIF 1585 kb)

Additional file 8: **Figure S8.** Percent change of amino acids of the roots under low N condition compared to control condition.The percentage was calculated according to (control-treatment)*100%/control. The seedlings of a newly formed hexaploid (neo-6*×*), its diploid (2*×*) and tetraploid (4*×*) parents, and natural allohexaploid (nat-6*×*) were subjected to low N condition (0.1 mM) for 31 days. (TIF 4448 kb)

Additional file 9: **Figure S9.** Effects of low N condition on the expression of nitrate transporter genes in a newly formed hexaploid (neo-6*×*), its diploid (2*×*) and tetraploid (4*×*) parents, and natural allohexaploid (nat-6*×*). The values are means of 3–5 biological replicates. Asterisks indicated significant difference (*t* test, *P* < 0.05) between control and low N-stressed plants for a given genotype. The means of any two of all four lines at the same N condition were compared using *t* test (*P* < 0.05), and means followed by different letters at the same N condition are significant. The seedlings were subjected to low N condition (0.1 mM) for 7 days. (TIF 1508 kb)

Additional file 10: **Table S1.** The wheat lines used in this work. (XLSX 11 kb)

Additional file 11: **Table S2**. Primers for q-RT-PCR analysis. (XLSX 11 kb)

## Abbreviations

Car: Carotenoid
Chl: Chlorophyll
*E*: Transpiration rate
GDH: Glutamate dehydrogenase
GO: Glycolate oxidase
GS: Glutamine synthetase
*g*_s_: Stomatal conductance
NR: Nitrate reductase
NRT: Nitrate transporter
*P*_N_: Net photosynthetic rate
