# Supplementary figures and images for: A newly formed hexaploid wheat exhibits immediate higher tolerance to nitrogen-deficiency than its parental lines
Source: BMC Plant Biol. 2018 Jun 7;18:113. doi: 10.1186/s12870-018-1334-1 (PMC5992729; doi:10.1186/s12870-018-1334-1)

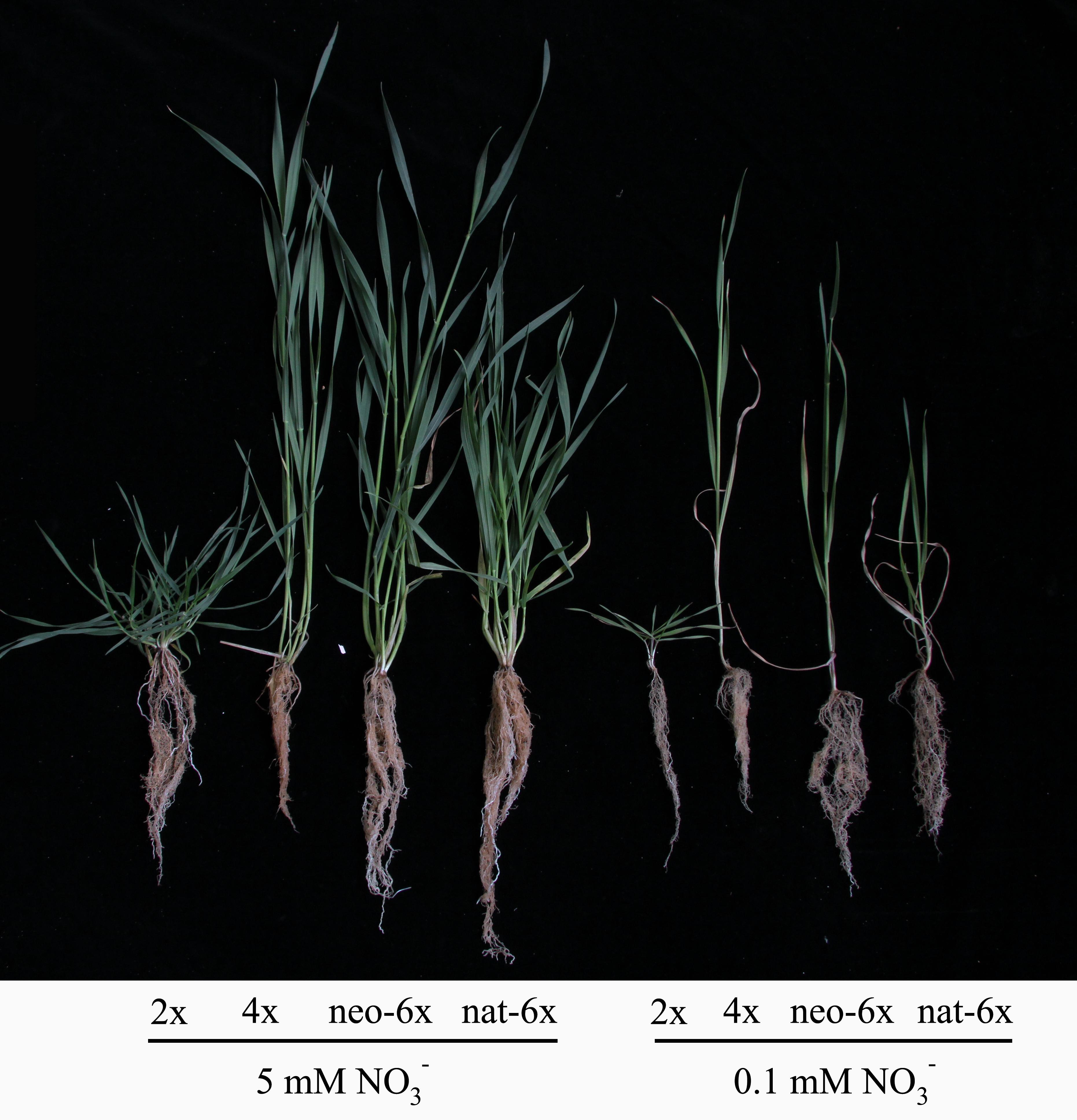

Supplement: Supplementary file 1 — Figure S1. Effects of low N condition on growth status of a newly formed hexaploid (neo-6×), its diploid (2×) and tetraploid (4×) parents, and natural allohexaploid (nat-6×). The seedlings were subjected to low N condition (0.1 mM) for 28 days. (JPG 749 kb) [file 12870_2018_1334_MOESM1_ESM.jpg]

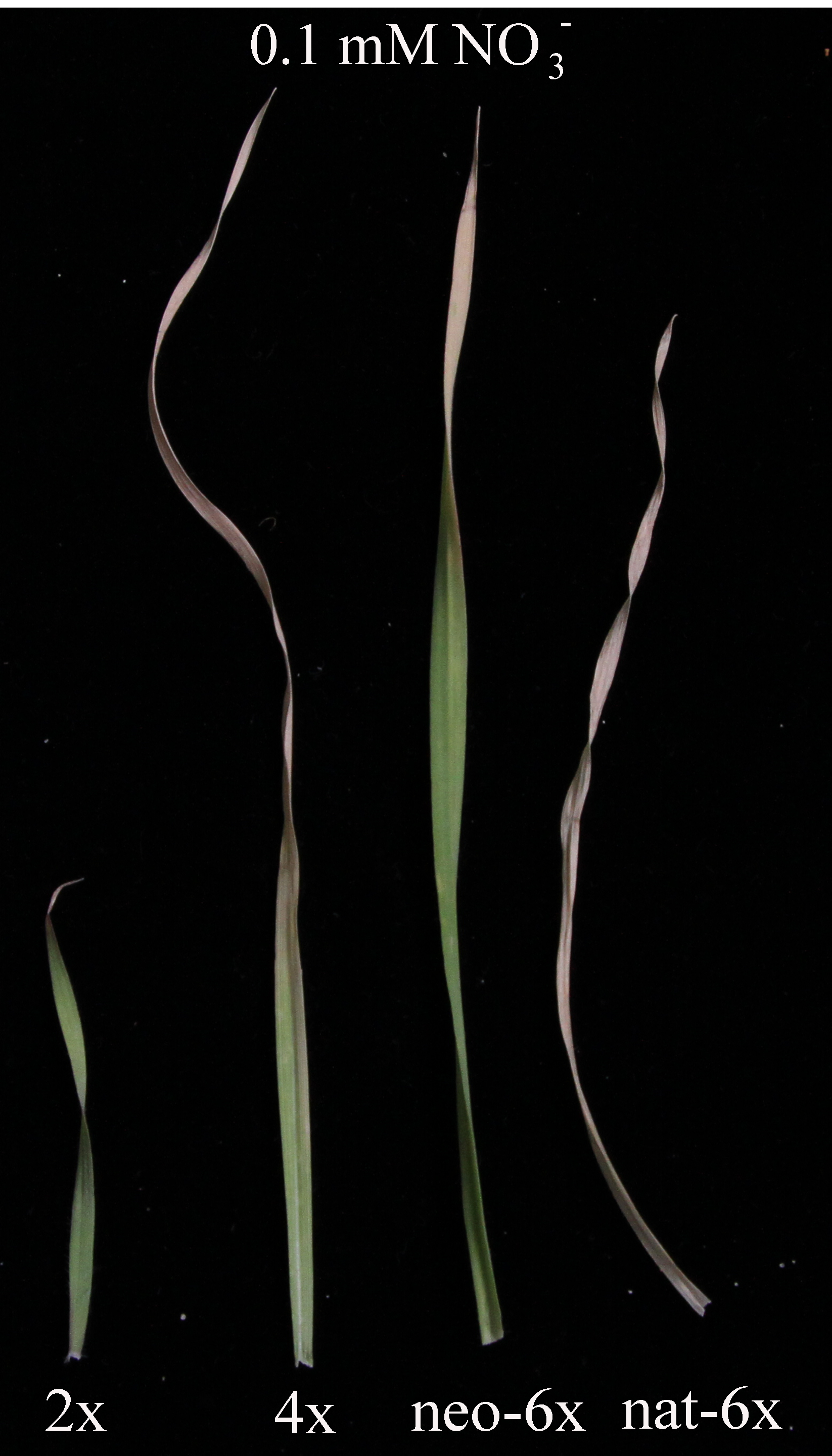

Supplement: Supplementary file 2 — Figure S2. Effects of low N condition on second leaf of a newly formed hexaploid (neo-6×), its diploid (2×) and tetraploid (4×) parents, and natural allohexaploid (nat-6×). The seedlings were subjected to low N condition (0.1 mM) for 28 days. Second leaf at below showed a clear difference among the four wheat lines under low N condition. (TIF 7026 kb) [file 12870_2018_1334_MOESM2_ESM.tif]

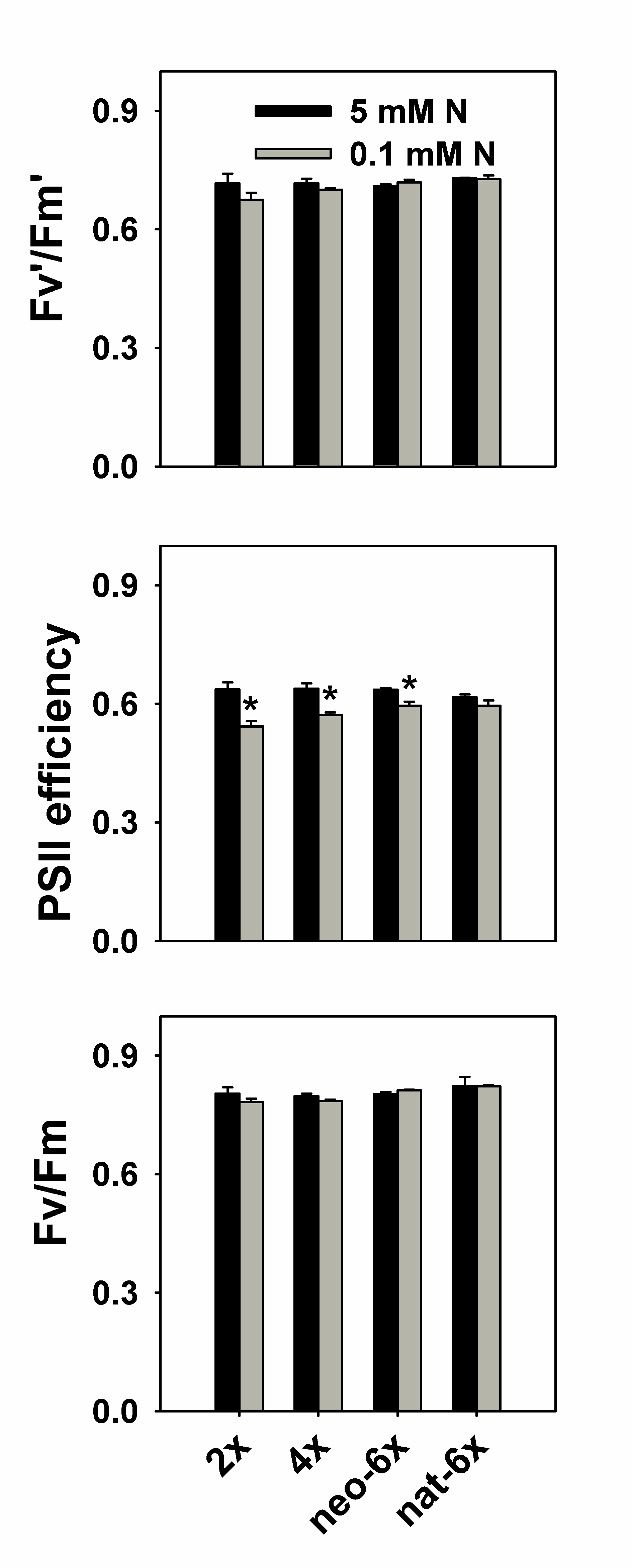

Supplement: Supplementary file 3 — Figure S3. Effects of low N condition on photosynthetic electron transport in a newly formed hexaploid (neo-6×), its diploid (2×) and tetraploid (4×) parents, and natural allohexaploid (nat-6×). The seedlings were subjected to low N condition (0.1 mM) for 31 days. Fv’/Fm′, efficiency of excitation capture by open PSII centers; Fv/Fm, maximum quantum yield of photosystem II. Asterisks indicated significant difference (t test, P < 0.05) between control and low N-stressed plants for a given genotype. (TIF 859 kb) [file 12870_2018_1334_MOESM3_ESM.tif]

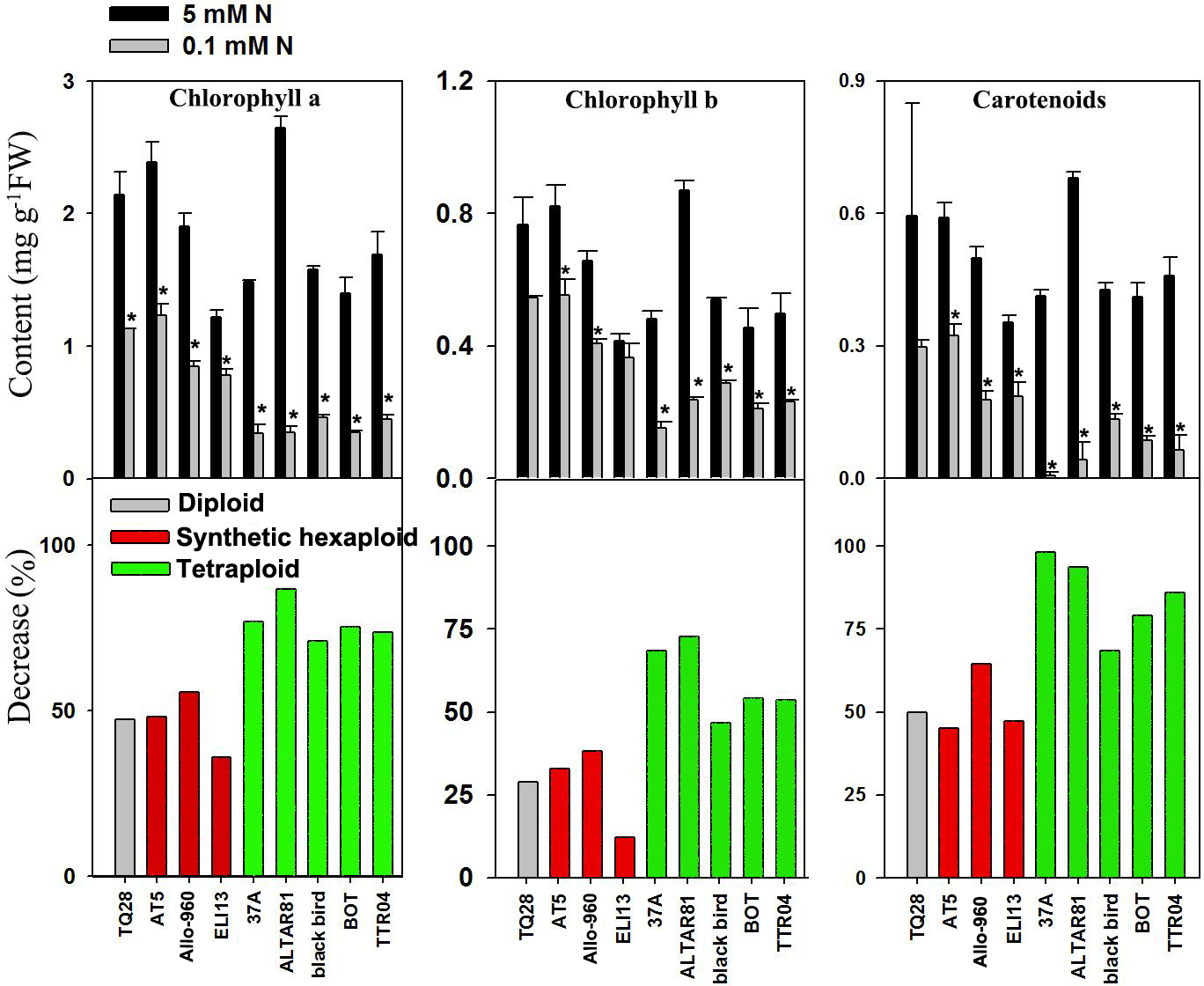

Supplement: Supplementary file 4 — Figure S4. Effects of low N condition on the chlorophyll and carotenoid contents of synthetic hexaploid wheats (BBAADD genome), diploid wheat (DD genome) and tetraploid wheats (BBAA genome). Diploid wheat:TQ18; newly formed (synthetic) hexaploid wheats: AT5, Allo-960 and ELI 13; tetraploid wheats: 37A, ALTAR81, black bird, BOT and TTR04. The seedlings were subjected to low N condition (0.1 mM) for 31 days. The values are means of three biological replicates. Asterisks indicated significant difference (t test, P < 0.05) between control and low N-stressed plants for a given genotype. (TIF 1779 kb) [file 12870_2018_1334_MOESM4_ESM.tif]

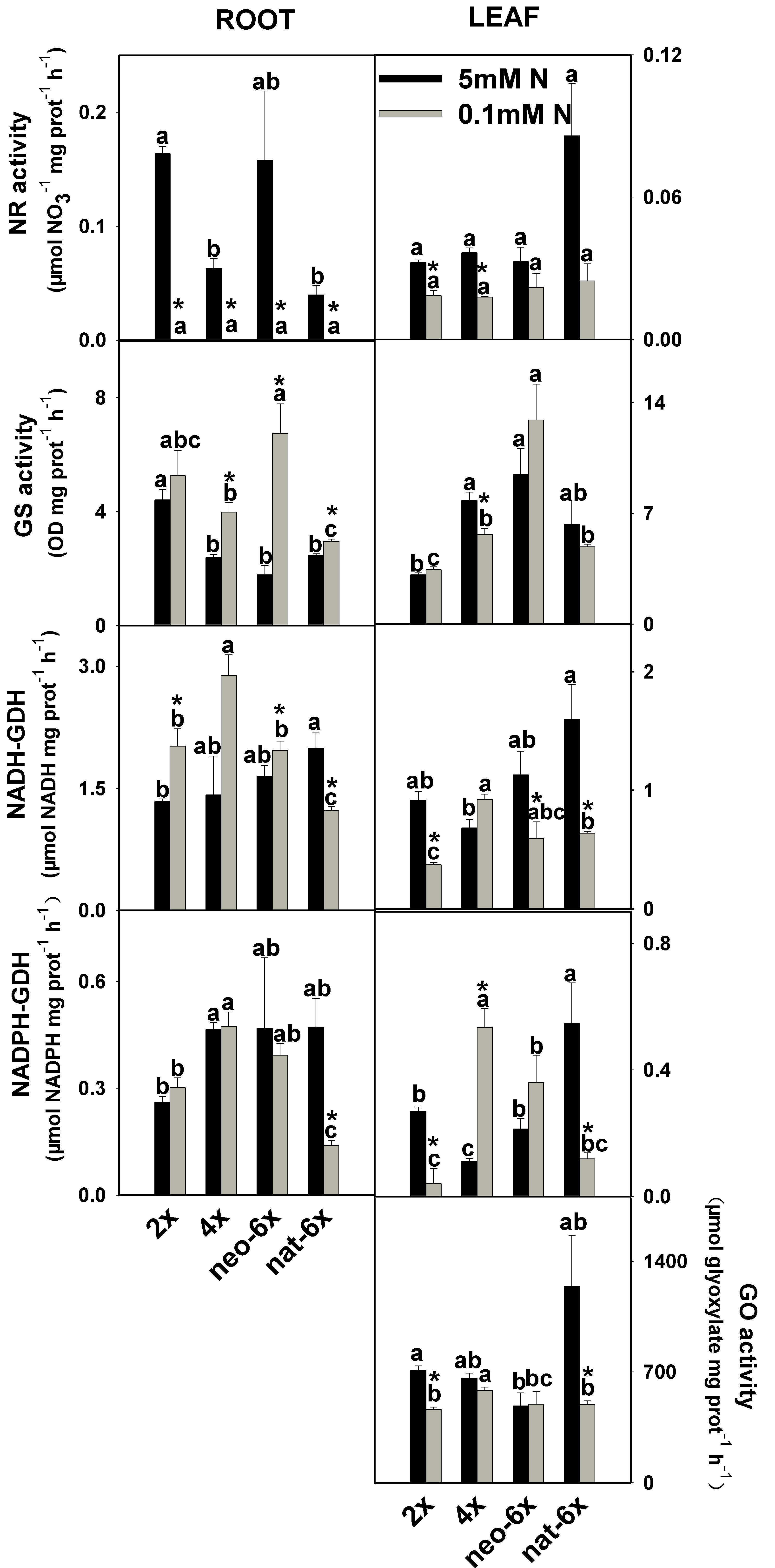

Supplement: Supplementary file 5 — Figure S5. Effects of low N condition on the activities of enzymes involved in nitrogen assimilation of a newly formed hexaploid (neo-6×), its diploid (2×) and tetraploid (4×) parents, and natural allohexaploid (nat-6×). The values are means of four biological replicates. Asterisks indicated significant difference (t test, P < 0.05) between control and low N-stressed plants for a given genotype. The means of any two of all four lines at the same N condition were compared using t test (P < 0.05), and means followed by different letters at the same N condition are significant. The seedlings were subjected to low N condition (0.1 mM) for 31 days. NR, nitrate reductase; GS, glutamine synthetase; GDH, glutamate dehydrogenase; and GO, Glycolate oxidase. (TIF 1582 kb) [file 12870_2018_1334_MOESM5_ESM.tif]

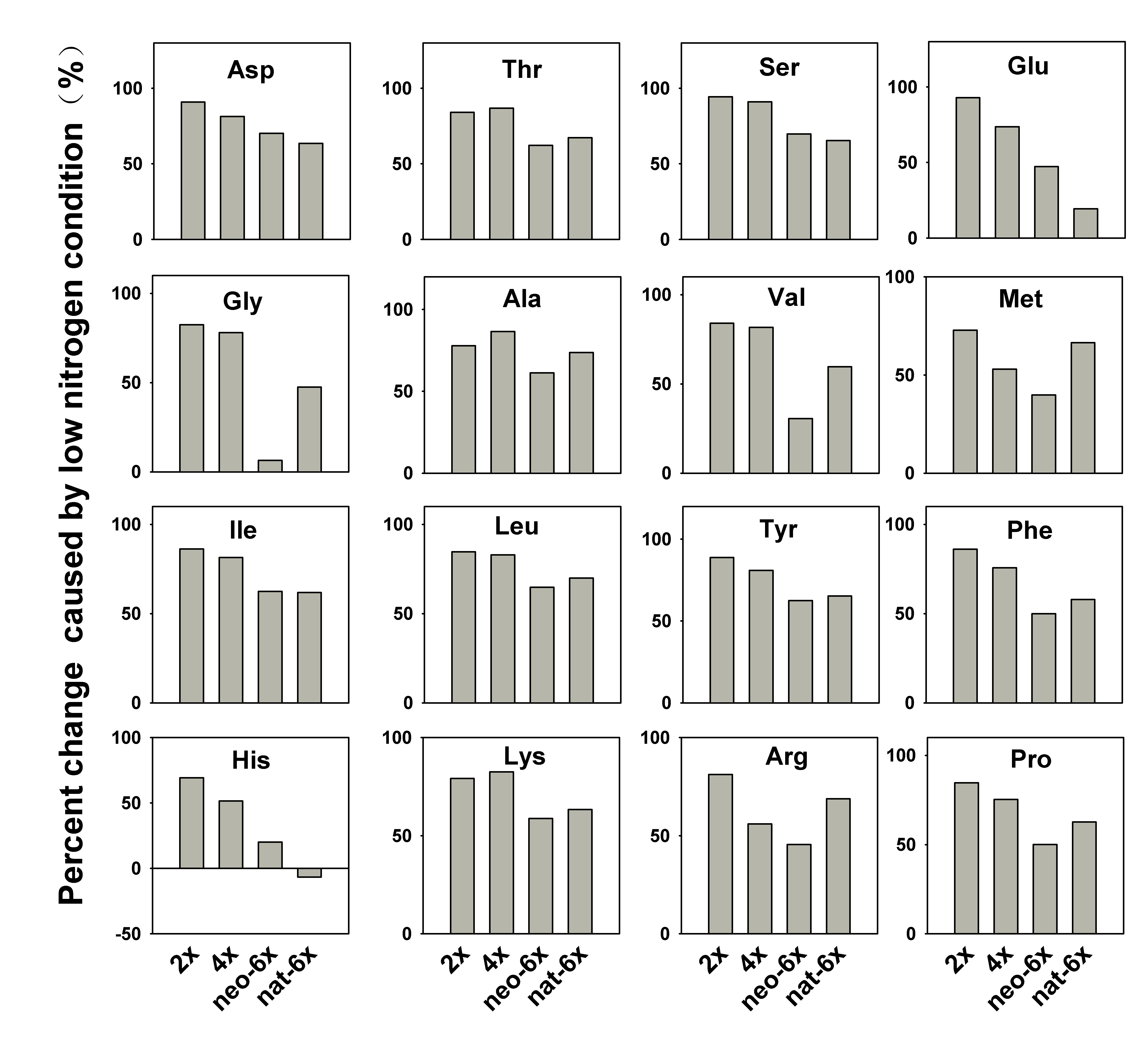

Supplement: Supplementary file 6 — Figure S6. Percent change of amino acids of the shoots under low N condition compared to control condition. The percentage was calculated according to (control-treatment)*100%/control. The seedlings of a newly formed hexaploid (neo-6×), its diploid (2×) and tetraploid (4×) parents, and natural allohexaploid (nat-6×) were subjected to low N condition (0.1 mM) for 31 days. (TIF 4881 kb) [file 12870_2018_1334_MOESM6_ESM.tif]

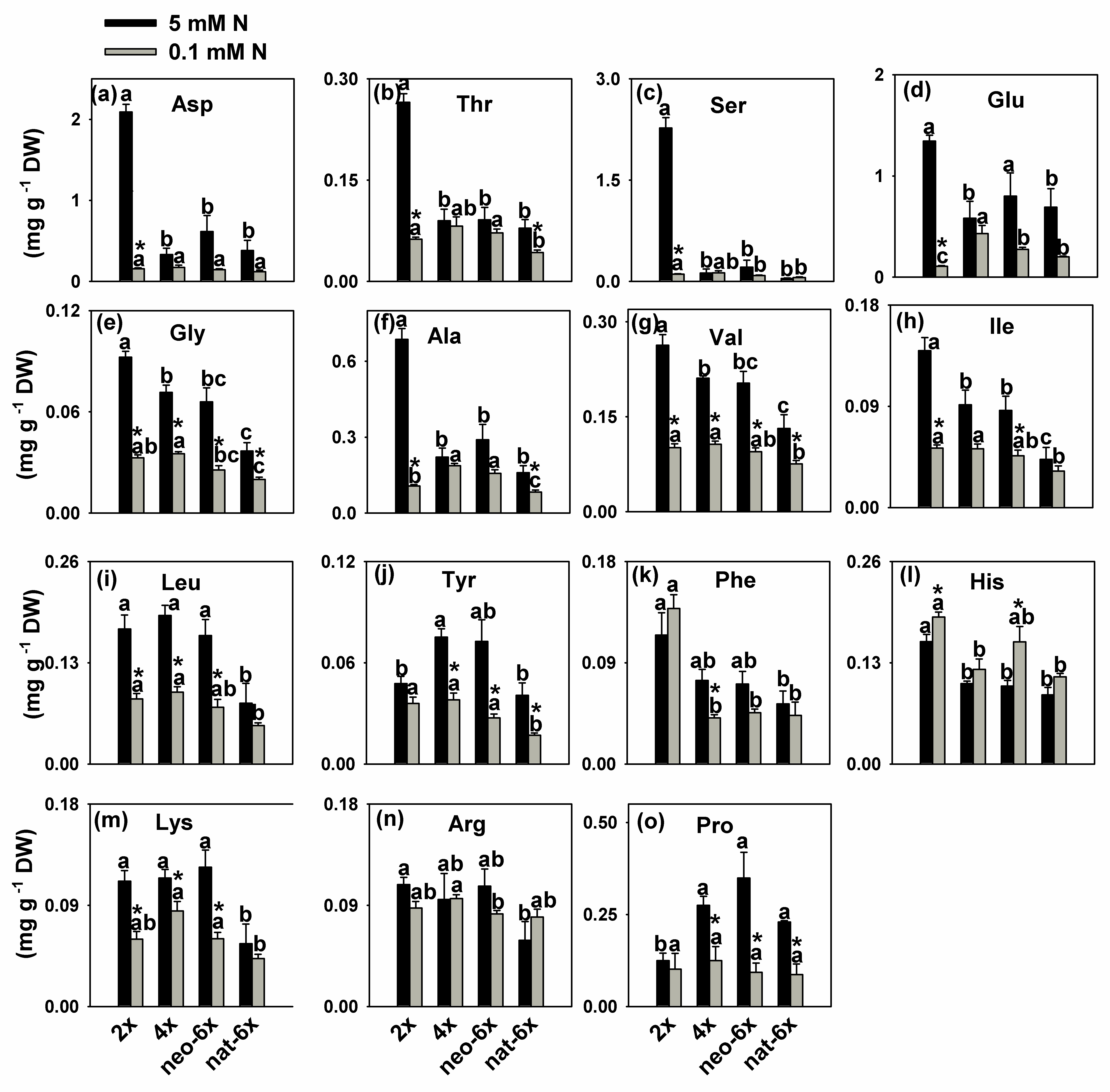

Supplement: Supplementary file 7 — Figure S7. Effects of low N condition on the contents of amino acids in roots of a newly formed hexaploid wheat (neo-6×), its diploid (2×) and tetraploid (4×) parents, and natural allohexaploid (nat-6×). The values are means of four biological replicates. Asterisks indicated significant difference (t test, P < 0.05) between control and low N-stressed plants for a given genotype. The means of any two of all four lines at the same N condition were compared using t test (P < 0.05), and means followed by different letters at the same N condition are significant. The seedlings were subjected to low N condition (0.1 mM) for 31 days. (TIF 1585 kb) [file 12870_2018_1334_MOESM7_ESM.tif]

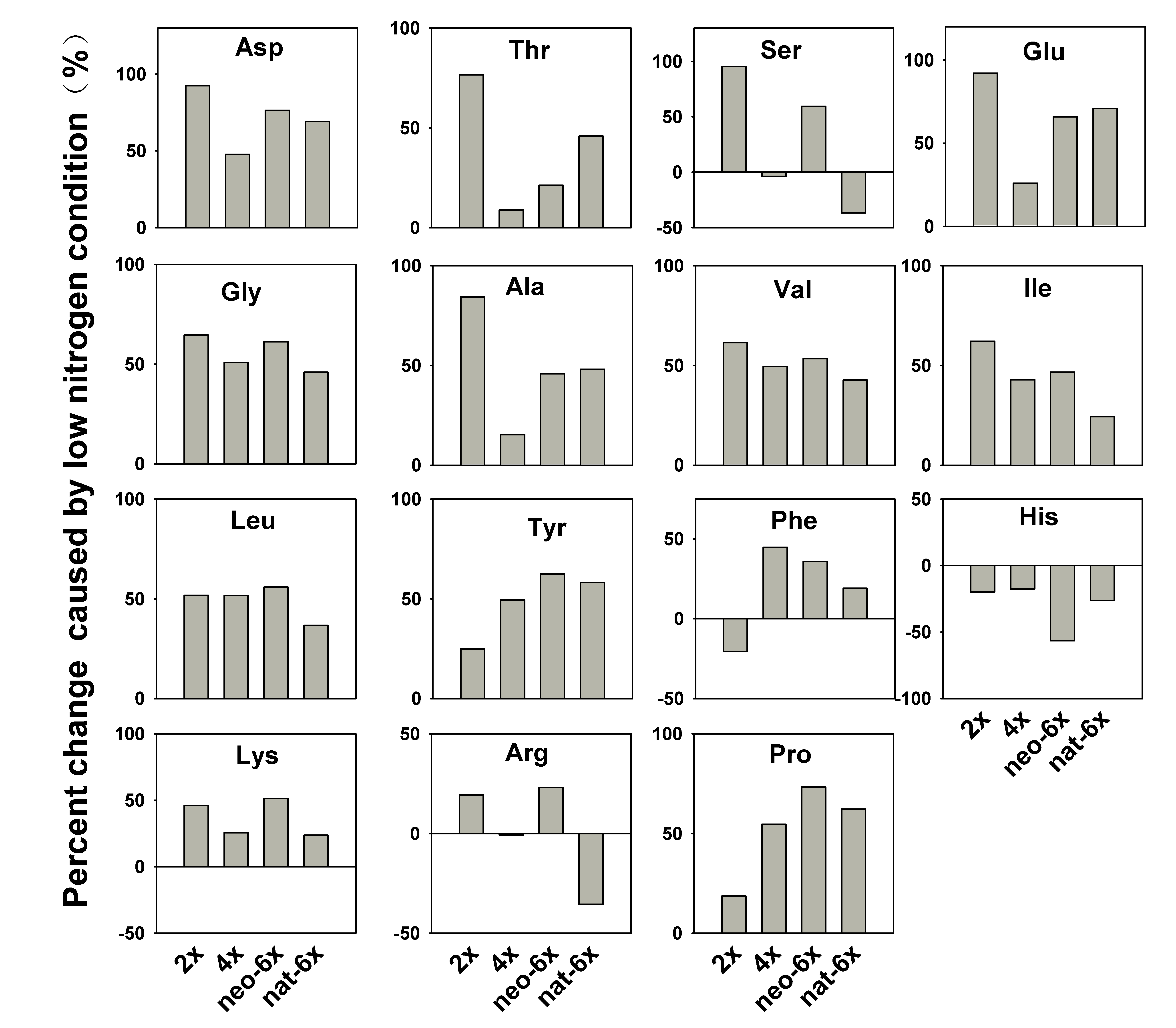

Supplement: Supplementary file 8 — Figure S8. Percent change of amino acids of the roots under low N condition compared to control condition.The percentage was calculated according to (control-treatment)*100%/control. The seedlings of a newly formed hexaploid (neo-6×), its diploid (2×) and tetraploid (4×) parents, and natural allohexaploid (nat-6×) were subjected to low N condition (0.1 mM) for 31 days. (TIF 4448 kb) [file 12870_2018_1334_MOESM8_ESM.tif]

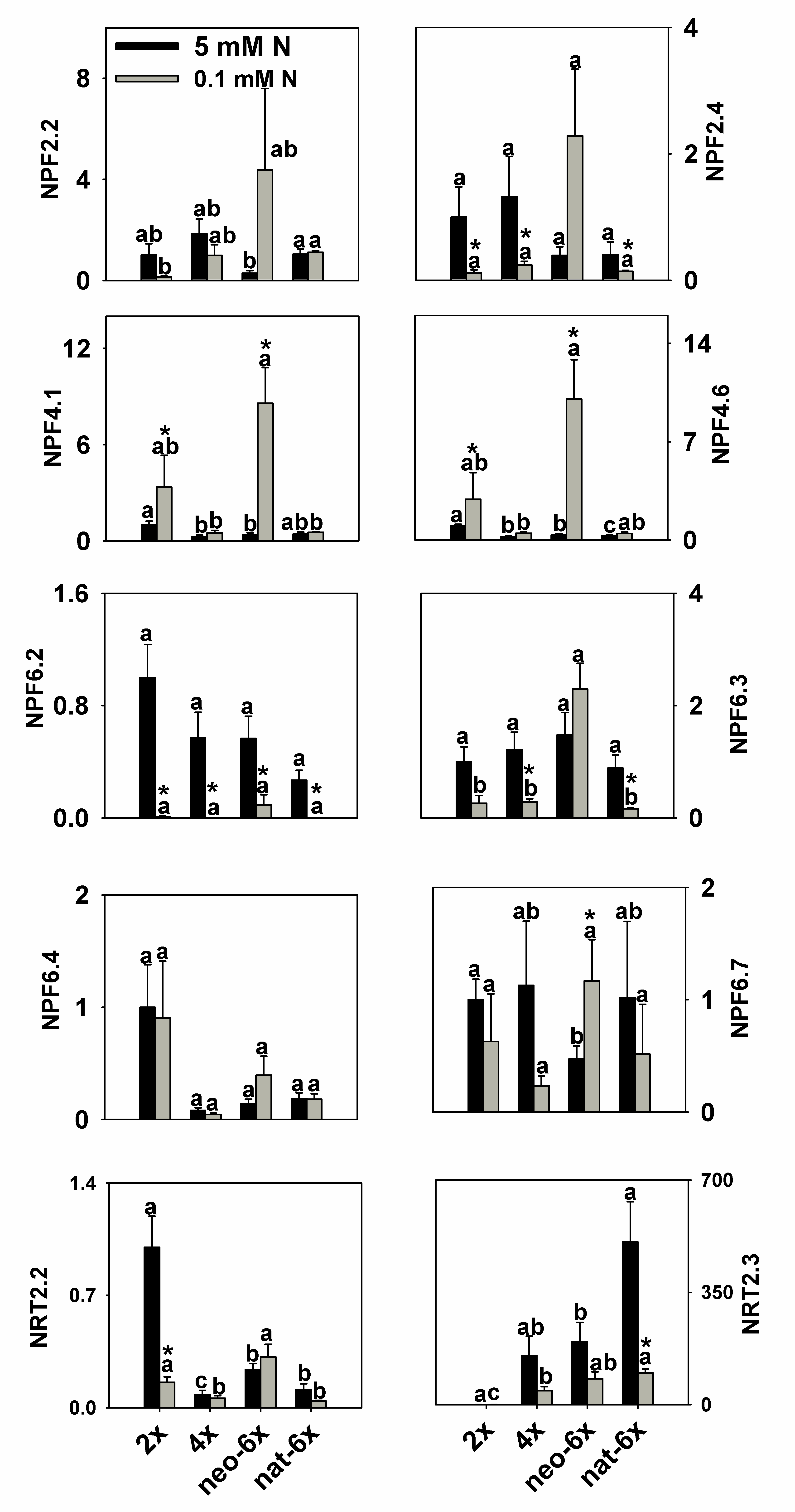

Supplement: Supplementary file 9 — Figure S9. Effects of low N condition on the expression of nitrate transporter genes in a newly formed hexaploid (neo-6×), its diploid (2×) and tetraploid (4×) parents, and natural allohexaploid (nat-6×). The values are means of 3–5 biological replicates. Asterisks indicated significant difference (t test, P < 0.05) between control and low N-stressed plants for a given genotype. The means of any two of all four lines at the same N condition were compared using t test (P < 0.05), and means followed by different letters at the same N condition are significant. The seedlings were subjected to low N condition (0.1 mM) for 7 days. (TIF 1508 kb) [file 12870_2018_1334_MOESM9_ESM.tif]
